# Clinical Application of Revised Laboratory Classification Criteria for Antiphospholipid Antibody Syndrome: Is the Follow-Up Interval of 12 Weeks Instead of 6 Weeks Significantly Useful?

**DOI:** 10.1155/2016/2641526

**Published:** 2016-08-17

**Authors:** Sang Hyuk Park, Seongsoo Jang, Chan-Jeoung Park, Hyun-Sook Chi

**Affiliations:** ^1^Department of Laboratory Medicine, Pusan National University College of Medicine, Pusan National University Hospital, Busan 49241, Republic of Korea; ^2^Biomedical Research Institute, Pusan National University Hospital, Busan 49241, Republic of Korea; ^3^Department of Laboratory Medicine, University of Ulsan College of Medicine and Asan Medical Center, Seoul 05505, Republic of Korea

## Abstract

*Background*. According to revised classification criteria of true antiphospholipid antibody syndrome, at least one of three antiphospholipid antibodies should be present on two or more occasions at least 12 weeks apart. However, it can be inconvenient to perform follow-up tests with interval of 12 weeks. We investigated clinical application of follow-up tests with interval of 12 weeks.* Method*. Totals of 67, 199, and 332 patients tested positive initially for the lupus anticoagulants confirm, the anti-*β*_2_ glycoprotein-I antibody, and the anti-cardiolipin antibody test, respectively, from Jan 2007 to Jul 2009. We investigated clinical symptoms of patients, follow-up interval, and results of each test.* Results*. Among patients with initial test positive, 1.5%–8.5% were subjected to follow-up tests at interval of more than 12 weeks. Among 25 patients with negative conversion in tests, patients with interval of more than 12 weeks showed clinical symptom positivity of 33.3%, which was higher than that of 12.5% with 6–12 weeks. Among 34 patients with persistent test positive, clinical symptoms positivity trended to be more evident in patients at interval of 6–12 weeks (47.4% versus 26.7%, *P* = 0.191) than more than 12 weeks.* Conclusion*. Less than 10% of patients with initial test positive had follow-up tests at interval of more than 12 weeks and the patients with persistent test positive at interval of more than 12 weeks showed trends toward having lower clinical symptoms than 6–12 weeks. More research is needed focused on the evidence that follow-up test at interval of more than 12 weeks should be performed instead of 6 weeks.

## 1. Introduction

Antiphospholipid antibody syndrome (APS) is an autoimmune disorder characterized by vascular thrombosis, complications during pregnancy, and the presence of antiphospholipid antibodies (APL) in plasma [1]. APL antibodies are measured using either a solid phase or a liquid phase test. In the solid phase test, an enzyme-linked immunosorbent assay (ELISA) is most widely used to detect various APL, including the anti-cardiolipin antibody (ACA), the anti-*β*_2_ glycoprotein-I (*β*_2_GPI) antibody, and the anti-prothrombin (aPT) antibody. In the liquid phase test, a clot-based functional assay detects APL in the form of the lupus anticoagulants (LA) associated with prolongation of phospholipid-dependent clotting time [2]. The patient is classified as true APS when at least one clinical sign or symptom and one positive laboratory test is identified. Currently, the LA, the IgG and/or IgM anti-*β*_2_GPI antibody and the IgG and/or IgM ACA laboratory tests are used for the classification of true APS.

In 1998, the Sapporo criteria were proposed for the classification of true APS. According to the Sapporo criteria, the patient is classified as true APS when at least one of the two clinical criteria is present and at least one of the two laboratory criteria is positive [3, 4]. Clinical criteria include the presence of vascular thrombosis or miscarriage, and the laboratory criteria include the presence of medium or high titers of the IgG or IgM ACA (using *β*_2_GPI-dependent ELISA methods) or the presence of the LA (based on International Society on Thrombosis and Haemostasis [ISTH] criteria), measured on two or more occasions at least 6 weeks apart.

In 2006, the revised Sydney criteria were proposed for the classification of true APS. In those criteria, the clinical criteria remained unchanged, but the laboratory criteria were revised [5]. The Sydney criteria added the IgG or IgM anti-*β*_2_GPI antibody test to the laboratory criteria and the follow-up interval was prolonged to 12 weeks.

However, it can be inconvenient practically for patients to undergo follow-up tests at interval of 12 weeks because it requires revisit to the clinic for APS suspected patients after relatively long time from initial evaluation. Therefore in our study, we evaluated clinical application of follow-up tests with interval of 12 weeks according to the Sydney criteria for the classification of true APS.

## 2. Materials and Methods

The LA screening and confirm test, the IgG or IgM *β*_2_GPI antibody test, and the IgG or IgM ACA test were requested on the total of 3,526 patients who were suspicious of APS by the clinicians. Samples of healthy subjects were excluded in our study population. All three tests (LA screening and confirm test, the IgG or IgM *β*_2_GPI antibody test, and the IgG or IgM ACA test) were performed at the same time when ordered in the same patient. Since either IgG/IgM ACA or IgG/IgM anti-*β*_2_GPI antibody test was requested rather than both in some patients, the total number of patients on whom LA confirm, the IgG or IgM anti-*β*_2_GPI antibody, and the IgG or IgM ACA test were performed was calculated to be 3,526, 2,394, and 2,948, respectively, from Jan 2007 to Jul 2009. We investigated positive rates, implementation of follow-up tests and follow-up interval of the LA confirm test, the IgG or IgM *β*_2_GPI antibody test, and the IgG or IgM ACA test performed. We also investigated clinical symptom included in the classification criteria of APS for patients with initial test positive in each test mentioned above. This study was carried out according to the ethical guidelines of Asan Medical Center and was approved by the Institutional Review Board of Asan Medical Center. Informed consent was obtained from all individual participants included in this study.

### 2.1. Testing Methods

#### 2.1.1. The LA Screening Test

For the LA screening, the aPTT test (PTT LA; Diagnostica STAGO, Asnieres, France) and the dRVVT test (DVVtest®10, American Diagnostica Inc., Stamford, USA) were performed according to the manufacturers' guidelines. A value more than two standard deviations above the mean reference level was considered positive. Since our present study included APL assay results obtained from 2007 to 2009 and the ISTH 2009 recommendations were not established at that time, the revised LA guidelines (2009) regarding the generation of LA assay cut-off value were not applied in this study [6].

#### 2.1.2. The Mixing and LA Confirm Tests

In cases with dRVVT screening test positive, the DVV confirmation test (DVVconfirm®5, American Diagnostica Inc.) was performed, according to the manufacturer's guidelines to confirm the LA positivity. In cases with PTT LA test positive, mixing test was performed at first and the confirmatory test (STACLOT-LA; Diagnostica STAGO, France) was performed later to confirm the LA positivity only in cases with prolonged clotting time in mixing test. The “positive” LA assay result was defined when all three tests (LA screening test, mixing test, and LA confirm test) show positive result. For the clot detection in the LA assays, the ACL-TOP (Instrumentation Laboratory, MA, USA) coagulation analyzer was used.

#### 2.1.3. The IgG or IgM Anti-*β*_2_GPI Antibody Test

The IgG or IgM anti-*β*_2_GPI test using REAADS® IgM, IgG Beta 2 Glycoprotein-I Semi-Quantitative test kits (Corgenix Inc., Broomfield, USA) was performed according to the manufacturer's guidelines. A reading of more than 20 GPL for IgG (previously validated to be above the normal 99th percentile) and a value of more than 20 MPL for IgM were considered as test positive.

#### 2.1.4. The IgG or IgM ACA Test

The IgG or IgM ACA test, performed using QUANTA Lite*™* ACA IgG, IgM III test kits (Inova Diagnostics, San Diego, USA), was performed according to the manufacturer's instructions. A reading of more than 21 GPL for IgG (previously validated as above the normal 99th percentile) and a value more than 21 MPL for IgM were considered as test positive.

### 2.2. Investigation of Follow-Up Results and Test Interval on Each Test in Patients with Initial Test Positive

We assessed positivity of all initial and follow-up tests, based on review of electronic medical records (EMR). For patients with initial test positive on each test, we investigated the implementation of follow-up tests and divided follow-up interval based on 6 weeks and 12 weeks, which was mentioned at the Sapporo criteria and Sydney criteria, respectively. The results were classified into those obtained at two follow-up intervals: (i) 6–12 weeks and (ii) more than 12 weeks.

### 2.3. Comparison of the Clinical Symptom Positivity in the Patients with Initial Test Positive with respect to Different Follow-Up Interval

All clinical symptoms, such as vascular thrombosis or spontaneous fetal loss, but not superficial venous thrombosis (thus, following the APS classification criteria) were recorded in the 59 patients with initial test positive on each combination of tests. Data of clinical symptoms were obtained by retrospective review of EMR.

These patients were further categorized into four groups according to two criteria, the follow-up test results (negative conversion and persistent positive), and the follow-up test intervals (6–12 weeks and more than 12 weeks). The proportion and clinical symptoms positivity of each patient group categorized as follow-up test results were compared separately with respect to the different follow-up test interval to evaluate the clinical relevance of follow-up interval of more than 12 weeks.

### 2.4. Statistical Analysis

Fisher's exact test was performed to compare the clinical symptoms positivity of each patient subgroup with respect to different follow-up test interval. The Mann-Whitney* U* test was performed to compare the levels of antibody between thrombotic and obstetric APS subgroup. For all analyses, tests were two-tailed and *P* values ≤0.05 were considered statistically significant. All calculations were performed using SPSS 13.0.1 for Windows (SPSS Inc., Chicago, IL, USA).

## 3. Results

### 3.1. Implementation of Follow-Up Tests on Each Test Item in the Patients with Initial Test Positive according to Different Follow-Up Interval

Among 3,526, 2,394, and 2,948 patients on whom the LA confirm, the IgG or IgM anti-*β*_2_GPI antibody, and the IgG or IgM ACA tests were performed, total of 67 (1.9%), 199 (8.3%), and 332 (11.3%) patients yielded initial positive results in the LA confirm, the anti-*β*_2_GPI antibody, and the ACA test, respectively. Among these patients with initial test positive in the LA confirm, the anti-*β*_2_GPI antibody, and the ACA test, there were a total of 67 cases [10 (14.9%), 24 (12.0%), and 33 (9.9%) in the LA confirm, the anti-*β*_2_GPI antibody, and the ACA test, resp.] in which the follow-up testing in each item was performed. Because some patients showed positivity in more than one test, the total number of patients yielding initial test positive on each combination of test item and performed follow-up test was 59 and the detailed results were as follows; 7 patients in LA confirm only, 19 patients in anti-*β*_2_GPI only, 26 patients in ACA only, 2 patients in both LA confirm and ACA, 4 patients in both anti-*β*_2_GPI and ACA, and 1 patient in all three tests.

The follow-up test was performed during the 6–12-week interval in 9 (90.0%), 7 (29.2%), and 23 patients (69.7%), respectively, in the LA confirm, the anti-*β*_2_GPI antibody, and the ACA test. And the follow-up testing was performed later than 12 weeks in 1 (10.0%), 17 (70.8%), and 10 patients (30.3%), respectively, in the LA confirm, the anti-*β*_2_GPI antibody, and the ACA test.

### 3.2. Comparison of the Clinical Symptom Positivity in the Patients with Initial Test Positive with respect to Different Follow-Up Interval

Table 1 summarizes the proportion and clinical symptoms positivity of each patient subgroup categorized with respect to follow-up test results (negative conversion and persistent positive) and different follow-up test interval (6–12 weeks and more than 12 weeks).

Among total 59 patients with initial test positive on each combination of test and on whom follow-up tests were performed at two different intervals, 25 (42.4%) patients showed negative conversion at follow-up test (16 patients with interval of 6–12 weeks and 9 patients with interval of more than 12 weeks) and 34 (57.6%) patients showed persistent positive results at follow-up test (19 patients with interval of 6–12 weeks and 15 patients with interval of more than 12 weeks).

Among 25 patients with negative conversion, patients with interval of more than 12 weeks were only nine, which was less than sixteen patients with interval of 6–12 weeks and also these patients showed clinical symptom positivity of 33.3%, which was higher than that of 12.5% in those with interval of 6–12 weeks (*P* = 0.230) although not statistically significant. Among 34 patients with persistent positive results, clinical symptoms positivity trended to be more evident in patients with interval of 6–12 weeks (47.4% versus 26.7%, *P* = 0.191) than more than 12 weeks. In 9 patients who showed persistent positive results at follow-up testing with interval of 6–12 weeks and also clinical symptom positive, all of them received another follow-up testing at later than 12 weeks after initial testing and all 9 patients showed positive results.

Among 18 patients (5 patients with negative conversion and 13 patients with persistent positivity) who showed clinical symptom positivity, 7 (38.8%) patients were thrombotic APS and 11 (61.2%) patients were obstetric APS. When the type and levels of antibodies were compared between two symptomatic APS subgroups, we found that the level of ACA tended to be lower in the obstetric APS subgroup than thrombotic APS subgroup (median 58.0 GPL and 51.0 MPL versus 71.0 GPL and 78.0 MPL, *P* = 0.198 and 0.123, resp.) but the differences were not statistically significant. The level of anti-*β*_2_GPI antibody and the type of detected antibodies did not show any significant differences between two patient subgroups.

## 4. Discussion

Previous studies have examined the association between persistent detection of APL and the presence of clinical symptoms. In the present work, we focused on the clinical usefulness of follow-up testing at interval of more than 12 weeks as recommended in the Sydney classification criteria of true APS, by analyzing the association between clinical symptom positivity and follow-up test interval in patients with initial test positive.

The current tests used for the classification of true APS have some limitations. First, we cannot detect all APL in single test. So we should perform multiple APL tests to avoid false negative. Second, with respect to the LA test, no standardized reference method addresses the issue of quality control, and no available technique can detect all LA. Third, the anti-*β*_2_GPI antibody and ACA tests are not associated with universally accepted criteria of positivity [7–9].

In the present study, only 9.9%–14.9% of patients who yielded positive APS laboratory test results underwent follow-up testing, and only 1.5%–8.5% of patients with positive APS laboratory test results were followed-up with interval of more than 12 weeks. This low rate of follow-up testing may be partly attributable to practical difficulties in performing follow-up tests, because most of our subjects were outpatients. To increase follow-up testing rates, the application of electronic program automatically notifying the need of follow-up testing to responsible physicians after predefined periods may be considered. And continuing education and persuasion to follow the recommended follow-up test interval also need to be performed for the clinicians. In addition, some clinicians may believe that low value of antibody strength and/or single positivity would be insufficient to warrant follow-up testing, and this may be also at least partly responsible for the low rate of follow-up testing.

The clinical importance of persistent LA, anti-*β*_2_GPI antibody, and ACA positive test results in the classification of true APS has been examined in several previous studies. Previous studies indicated that patients yielding two or more positive LA test results were at increased risk of vascular thrombosis and morbidity during pregnancy (with odds ratio [OR] of 6–10) [10–12]. Another study indicated that patients with two or more positive LA tests had an OR of 5.7–7.3 for vascular thrombosis [13]. In addition, another study showed that negative conversion at follow-up testing can be related to treatment, which suggests that the persistent APL assay results would be important indicator of “real symptomatic” APS [14]. Therefore, the association between persistent presence of LA and clinical symptoms of APS is well established compared with other test items that has been in conflict [15–21] and, in this study, the clinical symptom positivity rates among patients with persistent positivity in LA tests were 50.0% (1/2), which was higher than that of 23.1% (3/13) and 30.8% (4/13) in anti-*β*_2_GPI antibody test and ACA test, respectively. These results corresponded with those of previous literatures mentioned above.

In addition, a recent study demonstrated that the persistent APL assay results are observed more frequently in patients with triple positivity than those with double or single positivity at initial testing, which suggests that the high-risk patients with triple positive APL assay results are identified early at the time of initial screening tests [22]. These results emphasize the importance of multiple positivity at initial APL assays in the classification of true APS. Since our study results showed low frequency of follow-up testing and only a few patients were confirmed as symptomatic APS at follow-up testing (13 patients), these data could not be compared directly with previous data but we found that, in these patients, the majority of them (9 patients) showed positive results in more than one test at initial screening, which may underscore the suggestions of previous study [22].

The rationale for use of a longer follow-up interval given in the Sydney criteria is to increase specificity in the classification of true APS by avoiding misdiagnosing “transient positive” results as “true positives” [4]. A previous study demonstrated that the Sydney criteria may be superior to the Sapporo criteria by way of limiting the inclusion of a heterogeneous group of patients and also by way of providing a risk-stratified approach [23]. However, the increase of specificity in the classification of true APS and its clinical relevance in the Sydney criteria has not been adequately guaranteed, because a previous study reported that the sensitivity, specificity, positive predictive value, and negative predictive value of the Sapporo criteria in the classification of true APS would be also satisfactory, as 71%. 98%, 95%, and 88%, respectively [3]. In addition, another recent study also reported that although the Sydney criteria allow the inclusion of patients with anti-*β*_2_GPI antibody as isolated serologic marker, the wider follow-up intervals seem unlikely to make significant differences [24]. Our study results revealed that 33.3% of patients who showed negative conversion at follow-up interval of more than 12 weeks had clinical symptoms included in the classification criteria of true APS. This proportion of clinical symptoms positivity was higher than 12.5% of the patients at follow-up interval of 6–12 weeks. This result may suggest that 33.3% of patients who had possibilities of APS still can be misclassified as negative (transient positive) when the Sydney criteria are applied and this proportion is higher than 12.5% when the Sapporo criteria are applied, although the number of patients in each group was quite small and therefore the statistical power is limited. Notably, our study demonstrated that the clinical symptoms positivity of the patients who showed persistent positive APL test results at follow-up testing tended to be higher (47.4%) when the Sapporo criteria are applied than the Sydney criteria (26.7%). Given that patients with both clinical symptom and persistently positive in LA, anti-*β*_2_GPI antibody, or ACA are suspicious for APS patients, our results suggest that the positive predictive value of the Sapporo criteria for the classification of true APS may be higher than the Sydney criteria, which may support the suggestions of two previous study results [3, 24]. In addition, our study found that all 9 patients who showed persistent positive results at follow-up testing with interval of 6–12 weeks and clinical symptom positive also showed positive results in another follow-up testing performed at later than 12 weeks after initial testing. These results also support high positive predictive value of the Sapporo criteria. However as previously mentioned, our study has limited statistical power due to relatively small number of patients in each group under investigation. Therefore, our study results should be thought of only as the study which suggested a need of large-scale study focused on the convincing evidence that the Sydney criteria should be performed instead of the Sapporo criteria.

Our study has some limitations. As previously mentioned, our study was based on the retrospective review so that we could not clarify the exact reason why the follow-up test was not carried out frequently. Additionally, the number of patients in whom the follow-up test was performed was relatively small. This may be negatively influenced by the statistical power of our study and may be contributed to the lack of significance shown in our study results. Further study based on the larger population is firmly required to confirm the hypothesis mentioned in this study. Finally, because our study was designed to compare the clinical symptom positivity in APS patients classified from the Sapporo criteria (proposed at 1998) and the Sydney criteria (proposed at 2006) at the time when the Sydney criteria were released, our study was performed with samples obtained between 2007 and 2009 and more contemporary population was not included in the analysis. This point can also be an additional limitation of our study.

In conclusion, our present study showed that less than 10% of patients with initial test positive in three APL assays executed follow-up tests at interval of more than 12 weeks, and the patients with persistent positive results in three APL assays at interval of more than 12 weeks showed trends toward having lower clinical symptoms than those at interval of 6–12 weeks. Our present study did not show significant clinical advantage of the Sydney criteria over the Sapporo criteria, but the statistical power of our present study is limited but to low patient number. Far more APL assays-positive patients should be required to give any confirmatory results, and more research is needed focused on the identification of evidence justifying the performance of follow-up test at interval of more than 12 weeks instead of 6–12 weeks.

## Figures and Tables

**Table 1:** Assessment of the clinical symptom positivity in the 59 patients with initial test positive on each combination of test item with respect to different follow-up interval.

	Number of patients/number of patients with clinical symptoms (%)			
	Follow-up interval		Follow-up interval	
	6–12 weeks	More than 12 weeks	6–12 weeks	More than 12 weeks
	Negative conversion (positive → negative, *N* = 25)		Persistent positive (positive → positive, *N* = 34)	
LA confirm only (*N* = 7)	5/1 (20.0%)	0/0	2/1 (50.0%)	0/0
Anti-*β*_2_GPI only (*N* = 19)	1/0 (0.0%)	5/2 (40.0%)	3/2 (66.7%)	10/1 (10.0%)
ACA only (*N* = 26)	9/0 (0.0%)	4/1 (25.0%)	10/3 (30.0%)	3/1 (33.3%)
LA confirm + ACA (*N* = 2)	1/1 (100.0%)	0/0	1/1 (100.0%)	0/0
Anti-*β*_2_GPI + ACA (*N* = 4)	0/0	0/0	3/2 (66.7%)	1/1 (100.0%)
LA confirm + anti-*β*_2_GPI + ACA (*N* = 1)	0/0	0/0	0/0	1/1 (100.0%)

Totals (*N* = 59)	16/2 (12.5%)	9/3 (33.3%), *P* ^*∗*^ = 0.230	19/9 (47.4%)	15/4 (26.7%), *P* ^*∗*^ = 0.191

LA: lupus anticoagulants; *β*_2_GPI: *β*_2_ glycoprotein-I; ACA: anti-cardiolipin antibody.

^*∗*^ *P* values were obtained from Fisher's exact test.
